# Mast Cells Initiate the Recruitment of Neutrophils Following Ocular Surface Injury

**DOI:** 10.1167/iovs.17-23398

**Published:** 2018-04

**Authors:** Srikant K. Sahu, Sharad K. Mittal, William Foulsham, Mingshun Li, Virender S. Sangwan, Sunil K. Chauhan

**Affiliations:** 1 Schepens Eye Research Institute, Massachusetts Eye and Ear, Harvard Medical School, Boston, Massachusetts, United States; 2 L.V. Prasad Eye Institute, Bhubaneswar, Odisha, India; 3 Department of Ophthalmology, Beijing Hospital, National Center of Gerontology, Beijing, P.R. China; 4 L.V. Prasad Eye Institute, Hyderabad, India

**Keywords:** corneal injury, mast cell, neutrophil

## Abstract

**Purpose:**

The purpose of this study was to investigate the contribution of mast cells to early neutrophil recruitment during ocular inflammation.

**Methods:**

In a murine model of corneal injury, the epithelium and anterior stroma were removed using a handheld motor brush. Cromolyn sodium (2% in PBS) eye drops were administered topically for mast cell inhibition. In vitro, bone marrow–derived mast cells were cultured alone or with corneal tissue. The frequencies of CD45^+^ inflammatory cells, CD11b^+^Ly6G^+^ neutrophils, and ckit^+^FcεR1^+^ mast cells in the cornea were assessed by flow cytometry. mRNA expression of CXCL2 was evaluated by real-time PCR and protein expression by ELISA. β-Hexosaminidase assays were performed to gauge mast cell activation.

**Results:**

Neutrophil infiltration of the cornea was observed within 1 hour of injury, with neutrophil frequencies increasing over subsequent hours. Concurrent expansion of mast cell frequencies at the cornea were observed, with mast cell activation (assessed by β-hexosaminidase levels) peaking at 6 hours after injury. Evaluation of CXCL2 mRNA and protein expression levels demonstrated augmented expression by injured corneal tissue relative to naïve corneal tissue. Mast cells were observed to constitutively express CXCL2, with significantly higher expression of CXCL2 protein compared with naïve corneal tissue. Culture with harvested injured corneas further amplified CXCL2 expression by mast cells. In vivo, mast cell inhibition was observed to decrease CXCL2 expression, limit early neutrophil infiltration, and reduce inflammatory cytokine expression by the cornea.

**Conclusions:**

Our data suggest that mast cell activation after corneal injury amplifies their secretion of CXCL2 and promotes the initiation of early neutrophil recruitment.

Tissue injury provokes an acute inflammatory response that acts as a double-edged sword. Although critical to containing certain threats (e.g., microbial invasion), the effector mechanisms of innate immunity inflict damage on adjacent, healthy tissues.^1^ Neutrophils are key mediators of collateral tissue damage and are highly involved in the early phase of the immune response.^2^ However, the deleterious potential of neutrophils extends beyond collateral tissue damage, with these cells implicated in diverse pathologic conditions including autoimmunity, vascular diseases, and cancer.^3^ As such, there is significant interest in the mechanisms that initiate neutrophil recruitment, including the contribution of sentinel cells resident in local tissues such as mast cells.^3^

Mast cells are widely spread throughout tissues but are found in particularly high numbers at interfaces with the environment, such as the skin and mucosal surfaces.^4^ At the ocular surface, mast cells are abundant in the peripheral cornea, limbus, and conjunctiva.^5–7^ After activation, mast cells rapidly release preformed and preactivated inflammatory factors into the local surroundings.^8^ These granules are comprised of a plethora of constituents including cytokines (such as TNF-α), growth factors, amines, and enzymes (such as β-hexosaminidase).^8^ The role of mast cells in ocular allergy is well established; indeed, our current therapeutic strategies aim to either prevent mast cell activation or abrogate the effects of their secreted compounds.^5^ Although renowned for their role in allergy, mast cells have been implicated in a broad spectrum of physiologic and pathologic processes that are independent of IgE-mediated allergic immune resoponses.^9^ Indeed, mast cells have been shown to induce nonallergic inflammation in the gut, synovial membrane, and skin.^10–12^ However, the contribution of mast cells to the initiation of nonallergic inflammation at the cornea remains unclear.

In this study, we conducted a series of experiments to determine how mast cells influence the recruitment of neutrophils after corneal injury. The function of mast cells was investigated both in vitro and in vivo, using a well-characterized murine model of corneal injury.^13–17^ Owing to the low vascularity and the scarcity of resident immune cells in cornea, this model provides an excellent in vivo system to study the recruitment and function of immune cells. Our data demonstrate that injury leads to rapid recruitment of neutrophils to the cornea, with an attendant increase in mast cell activation observed. In vivo mast cell inhibition limited the early infiltration of neutrophils into the cornea and curtailed the acute inflammatory response.

## Materials and Methods

### Animals

Six- to 8-week-old male and female C57BL/6 wild-type mice (Charles River Laboratories, Wilmington, MA, USA) were used in these experiments. The protocol was approved by the Schepens Eye Research Institute Animal Care and Use Committee, and all animals were treated according to the ARVO Statement for the Use of Animals in Ophthalmic and Vision Research.

### Cell Culture Assays

Mast cells were generated by culturing bone marrow cells in the presence of IL-3 (10 ng/mL) and stem cell factor (SCF; 50 ng/mL) for 3 to 4 weeks with change of media every 3 to 4 days. This method of cell culture generates a mast cell population with more than 95% purity. For mast cell stimulation assays, mast cells were cultured either in medium alone or stimulated with injured corneas with or without transwell insert (1-μm pore size; Corning Inc., Corning, NY, USA) for 6 hours at 37°C. Injured corneas were harvested with limbus, and two corneas were cultured per well. Cells and supernatants were then harvested for further analysis for quantitation of β-hexosaminidase, CXCL1, and CXCL2 using the methods described below.

### Corneal Injury Model

Mice were deeply anesthetized, and corneal injury was created as previously described.^13–17^ Briefly, a 3-mm trephine was used to mark the central cornea in right eye. Using the tip of a handheld motor brush (Algerbrush II; Alger Company, Inc., Lago Vista, TX, USA), the corneal epithelium and anterior stroma were removed. On completion of the procedure, triple antibiotic ointment was applied to the injured eyes, and a subcutaneous injection of buprenorphine was administered to mice to minimize injury-induced pain. Corneal injury was conducted in the right eye of each mouse. The control eyes in our study (without injury) were harvested from naïve mice. To study the in vivo effect of mast cells, 3 μL 2% cromolyn sodium (Sigma-Aldrich Corp., St. Louis, MO, USA) eye drops were administered topically to eyes at four time points on the day of injury (which were −6, −3, and 0 hours and 3 hours after injury). Control injured mice were treated with PBS drops. After 6 hours, mice were euthanized, and corneas (including limbus) were harvested.

### Corneal Tissue Digestion

Single cell suspensions were prepared from corneas as described previously.^15^ Briefly, corneas were digested in RPMI media (Lonza, Walkersville, MD, USA) containing 2 mg/mL collagenase type IV (Sigma-Aldrich Corp.) and 2 mg/mL DNase I (Roche, Basel, Switzerland) for 45 minutes at 37°C, and subsequently filtered through a 70-μm cell strainer.

### Flow Cytometry

Single cell suspension was prepared and stained with fluorochrome-conjugated monoclonal antibodies. Cell surface staining was performed to evaluate frequencies of neutrophils using antibodies against CD11 and Ly6G and frequencies of mast cells using antibodies against c-Kit and FcεR1. Appropriate isotypes were used as antibody controls. Intracellular staining was performed to detect TNF-α expression (mean fluorescence intensity [MFI]) in mast cells. Stained cells were analyzed using LSR II flow cytometer (BD Biosciences, San Jose, CA, USA) and Summit software (Dako Colorado, Inc., Fort Collins, CO, USA). Antibodies and isotype controls were purchased from Biolegend (San Diego, CA, USA).

### Real-Time PCR

Total RNA was isolated using the RNeasy Micro Kit (Qiagen, Valencia, CA, USA) and reverse transcribed into cDNA using Superscript III (Invitrogen, Carlsbad, CA, USA). Quantitative real-time PCR was then performed using Taqman Universal PCR Mastermix and preformulated primers for murine CXCL1 (Mm04207460-M1), CXCL2 (Mm00436450-M1), IL-1β (Mm00434228_m1), TNF-α (Mm00443258-M1), and glyceraldehype-3-phosphate dehydrogenase (GAPDH, Mm99999915_gl) in a Mastercycler Realplex2 (Eppendorf, Hamburg, Germany). The results were analyzed by the comparative threshold cycle method and normalized to GAPDH as an internal control.

### β-Hexosaminidase Assays

The levels of β-hexosaminidase enzyme were estimated using the β-n-acetylglucosaminidase assay kit (Sigma-Aldrich), which is based on the hydrolysis of 4-Nitrophenyl *N*-acetyl-β-d-glucosaminide (NP-GlcNAc).^18^ Briefly, mast cells or cornea (with limbus) were lysed using 0.1% Triton X-100 (Sigma-Aldrich Corp.) for 20 minutes on ice. After centrifugation, supernatants were harvested and incubated with 0.1 mg/mL NP-GlcNAc (substrate) for 30 minutes at 37°C. Following this, the enzyme-substrate reaction was stopped with 5 mg/mL sodium carbonate. Absorbance was measured at 405 nm using SpectraMax Plus 384 Microplate Reader (Molecular Devices, San Jose, CA, USA). β-Hexosaminidase levels were evaluated using the formula: U/mL = (A_405_sample – A_405_blank) × 0.05 × 0.3 × DF/A_405_standard × time × volume of sample in milliliters.

### ELISA

Levels of CXCL2 were estimated in the supernatants harvested from mast cell cultures and corneal lysates using commercially available ELISA kits (R&D Systems, Minneapolis, MN, USA) as per the manufacturer's instructions. Corneal lysates were created by subjecting corneal tissue to three freeze-thaw cycles at −80°C/4°C, with centrifugation conducted at 10,000 rpm for 10 minutes.

### Statistical Analysis

Mann-Whitney *U* tests or unpaired two-tailed Student *t*-tests were used as appropriate to determine significance, which was set at *P* < 0.05. Data are presented as the mean ± SD. Results shown are representative of three independent experiments. Samples sizes were estimated on the basis of previous experimental studies on corneal injury and inflammation.^13–17^

## Results

### Neutrophil Infiltration of the Cornea Occurs Within Hours of Injury

**Figure 1:**
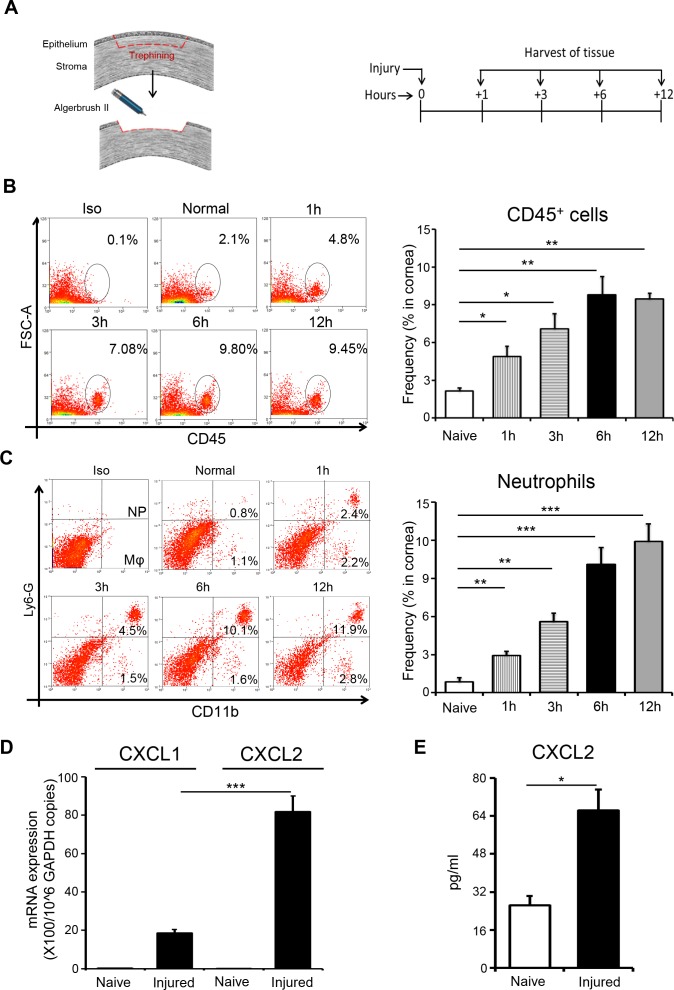
Corneal injury results in early recruitment of neutrophil to the ocular surface. (A) Schematic diagram depicting the mouse model of corneal injury used (left) and the time points at which tissues were harvested (right). (B) Representative flow cytometric dot plots (left) and cumulative bar chart (right) showing the frequencies of CD45^+^ inflammatory cells in the cornea at different time points after injury, relative to naïve mice. (C) Representative flow cytometric dot plots showing gating strategy for selecting CD11b^+^Ly6G^+^ neutrophils and CD11b^+^LyG^-^ macrophages in the cornea. Bar chart summarizes the frequencies of neutrophils in the cornea at different time points after injury, relative to naïve mice. (D) Bar chart depicting CXCL1 and CXCL2 mRNA expression at the ocular surface (normalized to GAPDH) in naïve and injured mice at 6 hours after injury, as quantified by real-time PCR. (E) Bar chart depicting CXCL2 protein expression at the ocular surface in naïve and injured mice at 6 hours after injury, as quantified by ELISA. Representative data from three independent experiments are shown and each experiment consisted of five animals. Data are represented as mean ± SD. *P < 0.05; **P < 0.01; ***P < 0.001.

To investigate the kinetics of inflammatory cell recruitment after corneal injury, we harvested corneas at different time points after injury and analyzed single cell suspensions of corneal tissue by flow cytometry (Fig. 1A). Noninjured corneas served as controls. Flow cytometric data reveal a progressive increase in the infiltration of CD45^+^ inflammatory cells into injured corneas relative to noninjured controls (Fig. 1B). Furthermore, our analysis demonstrated that the majority of the CD45^+^ population consisted of CD11b^+^Ly6G^+^ neutrophils (Fig. 1C). The CXC chemokine receptor 2-binding chemokines, CXC chemokine ligand 1 (CXCL1) and CXCL2, are potent chemoattractants that induce neutrophil recruitment.^3^ Therefore, we analyzed the expression of CXCL1 and CXCL2 mRNA in injured corneas compared with noninjured controls via real-time PCR. Our data demonstrate increased expression of CXCL1 and CXCL2 mRNA in injured corneas relative to controls (Fig. 1D). Furthermore, our data show that expression of CXCL2 mRNA was significantly higher than CXCL1 mRNA in injured corneas. The elevated expression of CXCL2 mRNA in injured corneas compared with naïve corneas was confirmed at the protein level, using ELISA performed on corneal lysates (Fig. 1E). Our results show that neutrophils infiltrate the cornea within hours of injury and indicate that corneal injury results in increased expression of the neutrophil chemoattractant CXCL2.

### Mast Cell Activation at the Cornea Occurs Within Hours of Injury

**Figure 2:**
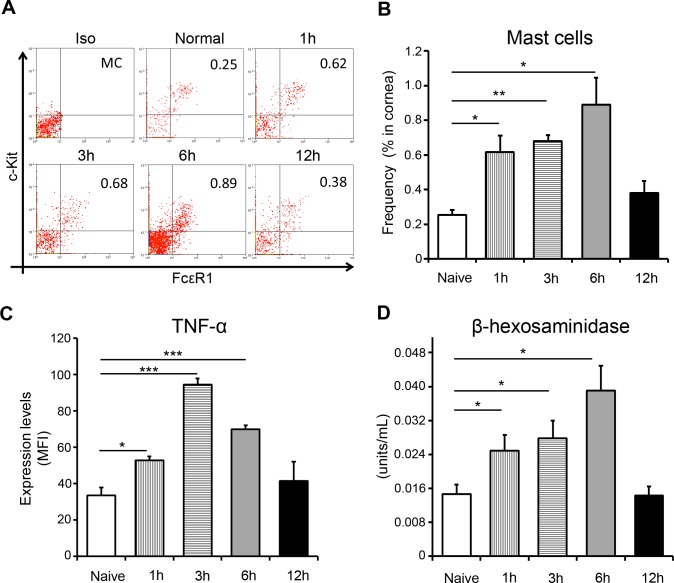
Corneal injury results in activation of mast cells at the ocular surface. (A) Representative flow cytometric dot plots and (B) cumulative bar chart showing the frequencies of ckit^+^FcεR1^+^ mast cells in the cornea at different time points after injury, relative to naïve mice. (C) Bar chart depicting the expression (MFI) of TNF-α by mast cells at different time points after injury, relative to naïve mice. (D) Corneal tissue (with limbus) were lysed, and β-hexosaminidase levels were estimated at different time points after injury, relative to naïve mice (as described in Materials and Methods). Representative data from three independent experiments are shown, and each experiment consisted of four to six animals. Data are represented as mean ± SD. *P < 0.05; **P < 0.01; ***P < 0.001.

Having observed increased neutrophil infiltration of the cornea at 1 hour after injury, we reasoned that such early recruitment of neutrophils must be driven by the local release of preformed proinflammatory mediators. Mast cells are present at the cornea and act as a repository for proinflammatory compounds; therefore, we hypothesized mast cell activation to be the event that initiates neutrophil recruitment.^5^ To investigate the kinetics of mast cells at the ocular surface, we harvested corneas (with limbus) at different time points after injury and enumerated the frequencies of ckit^+^FcεR1^+^ mast cells by flow cytometry (Figs. 2A, 2B). Our data show that the frequencies of mast cells had more than doubled at 1-hour after injury and progressively increased until 6 hours after injury, before declining to baseline at 12 hours after injury. To evaluate mast cell activation after corneal injury, we quantified expression of mast cell derived TNF-α and levels of β-hexosaminidase. TNF-α is an important marker of mast cell activation: it is an inflammatory cytokine that contributes to both innate and adaptive immunity, and functions in an autocrine manner to maintain the survival and activation of mast cells.^19,20^ The β-hexosaminidase assay is a widely adopted method for evaluating mast cell activation.^21^ Flow cytometric analysis demonstrated heightened expression of TNF-α by mast cells at 1, 3, and 6 hours after injury (Fig. 2C). Notably, by 12 hours after injury, expression of TNF-α had returned to levels comparable with those in naïve controls. Consistent with our TNF-α data, β-hexosaminidase assays conducted on corneal lysates demonstrated elevated levels of β-hexosaminidase at 1, 3, and 6 hours after injury relative to naïve controls, before returning to baseline at 12 hours after injury (Fig. 2D). Taken together, these data are highly suggestive of increased mast cell activation at the cornea following sterile injury.

### Mast Cells Constitutively Express High Levels of CXCL2

**Figure 3:**
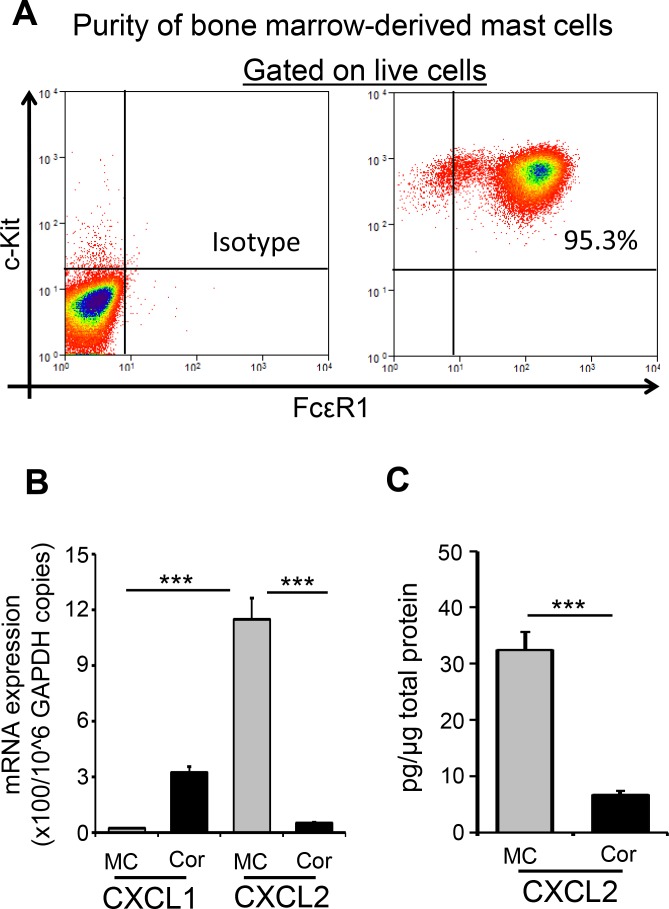
CXCL2 is constitutively expressed by mast cells. Mast cells were generated in vitro by culturing bone marrow cells in the presence of IL-3 and stem cell factor. (A) Flow cytometric dot plots demonstrating the purity of ckit^+^FcεR1^+^ mast cells derived from bone marrow cells. Mast cells and naïve corneas (Cor) were lysed to estimate (B) mRNA expression of CXCL1 and CXCL2 as quantified by real-time PCR and (C) protein expression of CXCL2 (normalized to per microgram of total protein) as quantified by ELISA. Two mice were used in each experiment, and each experiment was repeated three times. Data are represented as mean ± SD. ***P < 0.001.

The expression of chemokines CXCL1 and CXCL2 has been identified as an important mechanism in neutrophil recruitment.^3,22^ Having established that corneal injury increases both mast cell activation and neutrophil infiltration at the cornea, we sought to evaluate the expression of CXCL1 and CXCL2 by mast cells and compare this to expression by naïve corneal tissue. Because the cornea harbors very low frequencies of mast cells, we generated bone marrow–derived mast cells by culturing bone marrow cells in the presence of mast cell lineage cytokines, IL-3, and SCF (purity ≥ 95%) (Fig. 3A). Initially, we quantified the constitutive expression of CXCL1 and CXCL2 by mast cells and naïve corneas using real-time PCR (Fig. 3B). Mast cells exhibited significantly higher expression of CXCL2 relative to naïve corneas. Notably, CXCL1 was expressed at very low levels by mast cells compared with CXCL2. ELISA analysis of mast cells and corneal lysates corroborated the elevated expression of CXCL2 by mast cells, with higher expression of CXCL2 protein by mast cells compared with naïve corneas (Fig. 3C). These data show that mast cells constitutively express substantial levels of CXCL2.

### Injured Cornea Activates Mast Cells to Express Higher Levels of CXCL2

**Figure 4:**
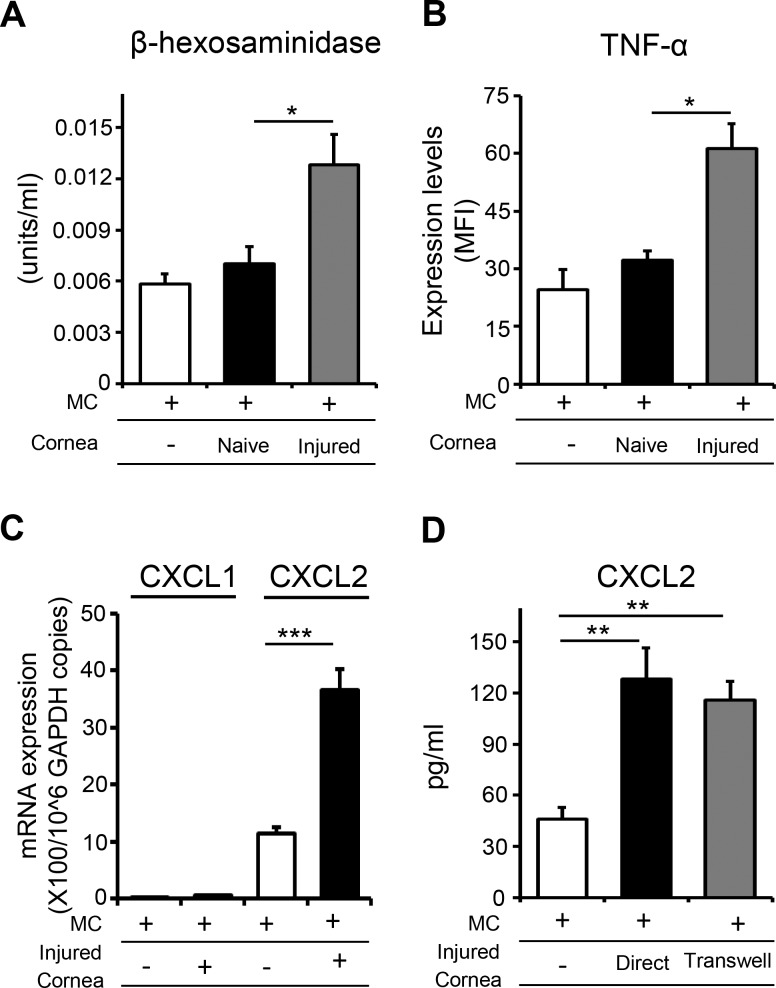
Injured corneas amplify the expression of CXCL2 by mast cells. Bone marrow–derived mast cells were cultured with injured corneas for 6 hours. Mast cells cultured alone and with naïve corneas served as controls. (A) Bar chart showing β-hexosaminidase levels in mast cells cultured with injured corneas, compared with mast cells cultured with naïve corneas. (B) Bar chart depicting the expression (MFI) of TNF-α by mast cells in the indicated groups, as quantified by flow cytometry. (C) Bar chart showing CXCL1 and CXCL2 expression by mast cells (normalized to GAPDH) in the indicated groups, as quantified by real-time PCR. (D) Protein levels of CXCL2 in culture supernatants of mast cells stimulated with injured corneas in the presence or absence of transwell inserts, as quantified by ELISA. Two mice were used in each experiment, and each experiment was repeated three times. Data are represented as mean ± SD. ***P < 0.05; **P < 0.01; ***P < 0.001.

To determine whether corneal injury amplifies chemokine expression by mast cells, we cultured mast cells with harvested injured corneas for 6 hours and evaluated mast cell activation and expression of CXCL1 and CXCL2. Control groups included mast cells cultured alone and mast cells cultured with naïve corneas**.** Culture with injured corneas resulted in increased mast cell activation relative to controls, as demonstrated by elevated expression of TNF-α and higher levels of β-hexosaminidase (Figs. 4A, 4B). Mast cells cultured with injured corneas exhibited significantly elevated CXCL2 mRNA expression relative to controls, as evaluated by real-time PCR (Fig. 4C). ELISA analysis of culture supernatants corroborated the elevated expression of CXCL2 by mast cells cultured with injured corneas, with higher levels of CXCL2 protein in the supernatant of mast cells cultured with injured corneas relative to the supernatant of mast cells cultured alone (Fig. 4D). Interestingly, the elevated secretion of CXCL2 that was observed when mast cells were cultured in direct contact with injured cornea was not abrogated when transwell inserts were included in the coculture system. Our data suggest that injured cornea upregulates mast cell CXCL2 expression via released soluble factors rather than contact-dependent mechanisms.

### In Vivo Blockade of Mast Cells Inhibits Early Neutrophil Recruitment and Corneal Inflammation After Injury

**Figure 5:**
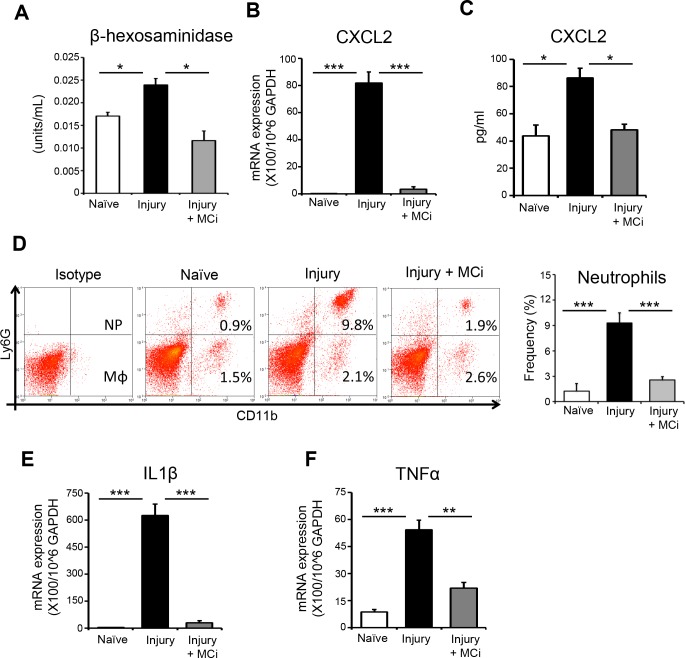
In vivo inhibition of mast cells prevent early recruitment of neutrophil and inflammation at ocular surface after corneal injury. Mast cell inhibitor (MCi) was topically administered to the ocular surface at −6, −3, 0, and 3 hours after corneal injury. Corneas with limbus were harvested at 6 hours after injury. PBS-treated mice with corneal injury and naïve mice served as controls. (A) Bar chart showing β-hexosaminidase levels in lysed corneas in MCi-treated mice, PBS-treated mice, and naïve controls. (B) Bar chart showing CXCL2 expression by mast cells (normalized to GAPDH) in the indicated groups, as quantified by real-time PCR. (C) Bar chart depicting protein levels of CXCL2 in the corneal lysate of indicated groups, as quantified by ELISA. (D) Single cell suspensions were prepared, and flow cytometry was performed. Representative flow cytometry plots (left) and bar chart (right) showing infiltration of CD11b^+^Ly6G^+^ neutrophils and CD11b^+^Ly6G^−^ macrophages to the ocular surface of indicated groups. (E) Bar chart showing expression of IL-1β mRNA in the conjunctivae harvested from indicated groups, as quantified by real-time PCR. (F) Bar chart showing expression of TNF-α mRNA in the conjunctivae harvested from indicated groups, as quantified by real-time PCR. Results are representative of three independent experiments. Each group consisted of five to six animals in each experiment. The values shown represent mean ± SD. *P < 0.05; **P < 0.01; ***P < 0.001.

Finally, using our in vivo model, we investigated the contribution of mast cells to the initiation of neutrophil recruitment and the promotion of corneal inflammation after injury. To determine this, we used a clinically relevant pharmacologic inhibitor, cromolyn sodium (MCi), to block mast cell activation.^23^ MCi was topically administered to the ocular surface at −6, −3, and 0 hours and 3 hours after corneal injury. Corneas were harvested at 6 hours after injury. PBS-treated mice with corneal injury and naïve mice served as controls. Treatment with MCi significantly inhibited mast cell activation after corneal injury, as demonstrated by reduced levels of β-hexosaminidase in the corneas of MCi-treated mice compared with PBS-treated injured controls (Fig. 5A). Notably, PBS-treated injured controls demonstrated elevated expression of CXCL2 mRNA relative to naïve mice, but treatment with MCi abrogated this effect (Fig. 5B). A similar trend of CXCL2 protein expression was detected by ELISA analysis of corneal lysates (Fig. 5C). Single cell suspensions of corneal tissue were prepared, and flow cytometry was performed. Corneal infiltration of CD11b^+^Ly6G^+^ neutrophils was significantly elevated in PBS-treated injured controls relative to naïve mice, but treatment with MCi repealed this effect (Fig. 5D). The cytokines IL-1β and TNFα have been shown to be elevated in ocular surface inflammation.^12–14,24^ Analysis of the expression of IL-1β and TNFα mRNA in our three groups by real-time PCR demonstrated significantly decreased expression of these proinflammatory cytokines in the corneas of MCi-treated mice compared with PBS-treated injured controls (Figs. 5E, 5F). Additionally, we tested the direct effect of MCi on neutrophil migration using in vitro chemotaxis assays. Our data showed no difference in the frequencies of migrated neutrophils to a CXCL2 gradient between MCi-treated and PBS-treated control groups (increase from baseline migration; 45.1 ± 6.6% compared with 40.7 ± 5.7%, respectively), suggesting that MCi does not directly modulate neutrophil migration. Collectively, our in vivo data demonstrate that mast cell inhibition results in decreased CXCL2 expression, with a concomitant reduction in early neutrophil infiltration of the cornea and diminished inflammation.

## Discussion

The collateral tissue damage meted out by neutrophils during corneal inflammation is potentially blinding. This study advances our understanding of the influence of mast cells in initiating early neutrophil recruitment at the ocular surface during acute inflammation. Specifically, our data show that (1) neutrophil infiltration of the cornea occurs within hours of injury and correlates with mast cell activation, (2) the constitutive expression of CXCL2 by mast cells is augmented by injured corneas, and (3) in vivo inhibition of mast cells suppresses expression of CXCL2 at the inflamed cornea and results in (4) decreased neutrophil infiltration and reduced corneal inflammation.

Neutrophils have historically been understood as the foot soldiers of innate immunity, performing a limited set of proinflammatory functions. Recently, we have begun to appreciate the more subtle contributions of these cells to both innate and adaptive immunity, as well as to chronic inflammatory conditions including autoimmunity, vascular diseases, and cancer.^3^ Given the important role of neutrophils in a broad spectrum of physiologic and pathologic processes, both the kinetics and the mechanisms governing neutrophil recruitment have attracted attention.^3,25^ Neutrophil kinetics have been reported in a wide range of organs including the liver, spleen, and bone marrow.^26^ Neutrophil accumulation in the dermis following sterile injury has been reported within 15 minutes.^27^ At the ocular surface, there is increased infiltration of the cornea with neutrophils during inflammation.^28,29^ However, to our knowledge, the kinetics of neutrophil migration to the cornea at early time points following injury have not yet been systematically investigated. In the present study, we demonstrate increased neutrophil infiltration of the cornea at 1 hour after injury. Based on this observation, we postulated that such rapid recruitment of neutrophils must be driven by the release of preformed proinflammatory mediators and identified mast cell activation as a plausible initiating event.

Our data correlate neutrophil recruitment and mast cell activation at early time points following sterile injury. Neutrophils respond to the potent chemoattractants CXCL1 and CXCL2, which have previously been shown to be expressed by a variety of cells including monocytes, macrophages, and dendritic cells.^30^ Given our hypothesis that mast cells are the predominant source of neutrophil chemoattractants at early time points following corneal injury, and considering the low frequencies of mast cells observed in noninflamed ocular surface tissues, we predicted low levels of CXCL1 and CXCL2 expression by naïve corneal tissue. Indeed, our findings demonstrate negligible mRNA and low protein expression of CXCL1 and CXCL2 by naïve corneas. In contrast, our data show that CXCL2 is constitutively expressed at high levels by mast cells, and moreover, we report that corneal injury amplifies this expression.

In addition to increased CXCL2 expression by mast cells after injury, we found increased levels of TNF-α expression. Interestingly, it has previously been shown that mast cell-derived TNF-α is critical for CXCL2-induced neutrophil adhesion to endothelial cells.^31^ It is important to note that there are other potential sources of neutrophil chemoattractants at the cornea; indeed, macrophages express CXCL1 and CXCL2,^22^ but we did not observe a significant infiltration of CD11b^+^Ly6G^−^ macrophages at early time points after injury. However, it is plausible that mast cells both promote neutrophil recruitment directly through increased expression of CXCL2 and indirectly through the release of proinflammatory mediators such as TNF-α and the subsequent activation of resident macrophages.

Cromolyn sodium is a clinically widely used mast cell inhibitor.^23^ The 2% cromolyn sodium eye drops are an effective treatment for allergic conjunctivitis.^32^ In the translational arm of our study, we evaluated the in vivo effect of mast cell inhibition with cromolyn sodium on nonallergic corneal inflammation. Treatment with a topical mast cell inhibitor was observed to significantly reduce corneal infiltration of neutrophils and decrease the expression of the proinflammatory cytokines IL-1β and TNF-α after injury relative to PBS-treated controls. Given that IL-1β is secreted by neutrophils during the inflammatory response,^33^ the observation of decreased IL-1β expression provides functional evidence for diminished neutrophil recruitment after mast cell inhibitor treatment. In light of the observed effect of cromolyn sodium treatment on the suppression of neutrophil infiltration, we conducted further experiments that demonstrated that cromolyn sodium does not directly regulate neutrophil migration.

## Conclusions

In summary, our data demonstrate that neutrophils are recruited rapidly to the cornea following injury, and this early infiltration is associated with increased mast cell activation. We identified that the important neutrophil chemoattractant CXCL2 is constitutively expressed by mast cells and that its expression is upregulated following ocular injury. Finally, we report that inhibition of mast cells at the ocular surface decreases expression of CXCL2 at injured corneas, suppresses neutrophil infiltration, and limits corneal inflammation.
